# Infectious Sporozoites of *Plasmodium berghei* Effectively Activate Liver CD8α^+^ Dendritic Cells

**DOI:** 10.3389/fimmu.2018.00192

**Published:** 2018-02-08

**Authors:** Rajesh Parmar, Hardik Patel, Naveen Yadav, Ritika Parikh, Khyati Patel, Aditi Mohankrishnan, Vishakha Bhurani, Urja Joshi, Sarat Kumar Dalai

**Affiliations:** ^1^Institute of Science, Nirma University, Ahmedabad, India

**Keywords:** CD8α^+^ dendritic cells, liver-stage immunity, memory CD8^+^ T cells, RAS, RNA-seq, toll-like receptor, interferon

## Abstract

Immunization with radiation-attenuated sporozoites (RAS) shown to confer complete sterile protection against *Plasmodia* liver-stage (LS) infection that lasts about 6 to 9 months in mice. We have found that the intermittent infectious sporozoite challenge to immune mice following RAS vaccination extends the longevity of sterile protection by maintaining CD8^+^ T cell memory responses to LS infection. It is reported that CD8α^+^ dendritic cells (DCs) are involved in the induction of LS-specific CD8^+^ T cells following RAS or genetically attenuated parasite (GAP) vaccination. In this study, we demonstrate that CD8α^+^ DCs respond differently to infectious sporozoite or RAS inoculation. The higher accumulation and activation of CD8α^+^ DCs was seen in the liver in response to infectious sporozoite 72 h postinoculation and found to be associated with higher expression of chemokines (CCL-20 and CCL-21) and type I interferon response *via* toll-like receptor signaling in liver. Moreover, the infectious sporozoites were found to induce qualitative changes in terms of the increased MHCII expression as well as costimulatory molecules including CD40 on the CD8α^+^ DCs compared to RAS inoculation. We have also found that infectious sporozoite challenge increased CD40L-expressing CD4^+^ T cells, which could help CD8^+^ T cells in the liver through “licensing” of the antigen-presenting cells. Our results suggest that infectious sporozoite challenge to prior RAS immunized mice modulates the CD8α^+^ DCs, which might be shaping the fate of memory CD8^+^ T cells against *Plasmodium* LS infection.

## Introduction

Malaria still remains a serious challenge to human health as millions of lives are at the risk of infection, particularly the children residing in South-East Asia and Sub-Saharan Africa (1). Tremendous efforts have been made for development of effective vaccines, but only RTS,S (Mosquirix), a subunit pre-erythrocytic stage malaria vaccine, has gone through phase III clinical trial. Despite respectable efficacy, the duration of protection conferred by RTS,S vaccine is very short, less than 6 months (2, 3). The radiation-attenuated sporozoites (RAS) vaccination has been very promising in inducing complete sterile immunity against liver-stage (LS) infection (4–6). However, the protection is still short lived among rodents as well as humans in spite of receiving multiple frequent doses of RAS (7–9). Similarly, repeated immunizations with genetically attenuated parasite (GAP) has shown the maintenance of only partial sterile protection (20–40%) (10). Interestingly, we have shown that the protection could be extended from 6 to 18 months in mice by the intermittent challenge with infectious sporozoites (Inf. Spz) (11) (manuscript in preparation). It is also reported that people living in endemic area maintain immunity following repeated exposure to malaria parasite (12, 13). The results obtained from experimental models including ours demonstrate that protection is dependent upon the presence of effector CD8^+^ T cells in the liver (14–19). Moreover, we have shown that the loss of CD8^+^ T cells in the liver of RAS immune mice is rescued following the Inf. Spz challenge. It has been shown that host (rodent as well as human) immunized with Inf. Spz under the cover of chloroquine are protected up to 28 months (20, 21). The above findings are indicative of the fact that infectious status of sporozoites may be playing a key role in modulating CD8^+^ T cell response that ensue longer-lived protection against *Plasmodia* LS infection.

It is a fact that the kind of danger signals host perceives from the pathogen would dictate the nature of innate immune response. The infectious status of sporozoites might influence the innate immune cells that ultimately modulate the CD8^+^ T cell response. Dendritic cells (DCs) are shown to be involved in the induction of protective immunity against various pathogens including *Plasmodia* (22, 23). Only limited studies demonstrated that the role of distinct subsets of DCs in the generation of malaria protective CD8^+^ T cells (22) including LS-specific CD8^+^ T cells, known to confer the sterile immunity evoked by RAS immunization (22). While depletion of DCs fails to induce protection induced by RAS vaccination, adoptive transfer of DCs loaded with circumsporozoite protein (CSP) antigen is shown to generate antigen-specific CD8^+^ T cells conferring partial protection on the challenge with Inf. Spz (24).

In case of *Plasmodia* LS infection, liver CD8α^+^ DCs have been shown to play an instrumental role in provoking immunity against LS *Plasmodia* infection (16, 25–27). Present study corroborates our findings wherein infectious status of sporozoite is shown to play a pivotal role in developing long-lasting protective sterile immunity against LS *Plasmodia* infection. We have characterized DCs in the liver and different lymphoid organs [spleen and liver-draining lymph nodes (LNs)], and looked for their activation status in response to Inf. Spz. Furthermore, we also found that Inf. Spz modulates the qualitative changes in the LS-specific CD4^+^ T cells as well as CD8^+^ T cells. We found that the infectious nature of sporozoites drives the accumulation and activation of CD8α^+^DCs in the liver and promotes type I interferon synthesis as well as higher expression of costimulatory molecules including CD40. The characteristics of CD8α^+^ DCs in the liver of Inf. Spz challenged mice indicate their involvement in modulation of LS-specific memory CD8^+^ T cells ensuring longer-lived protection. Upon investigating the possible role of CD4^+^ T cells in this process, we found that Inf. Spz challenge following RAS priming favored the generation of CD4^+^ T cells having upregulated CD40L (CD40 ligand) that might be helping license the DCs to promote longer-lived CD8^+^ T cell response.

## Materials and Methods

### Mice

Female C57BL/6 mice (4–8 weeks old) were brought from Zydus Research Center, Ahmedabad, Gujarat, India. Mice were housed at the central facility of Nirma University and handled as per the institutional animal handling guidelines. All animal experiments were reviewed and approved by the Institutional Animal Ethical Committee of Nirma University (CPCSEA Reg. No: 883/PO/ReBi/S/05/CPCSEA).

### Sporozoites Preparation

*Plasmodium berghei* (Pb) (ANKA strain) sporozoites were isolated from salivary glands by dissecting the infected *Anopheles stephensi* mosquitoes on day 18 after feeding with infected blood. The purified sporozoites were attenuated by exposing to 12,000 rads of gamma-radiation (Bhabhatron) in the facility of BV Patel PERD Center, Ahmedabad. All experiments were reviewed and approved by the Institutional Biosafety Committee (IBSC) of Institute of Science, Nirma University (IBSC Reg. No: UA3K1PKH).

### Immunization

#### Assessment of Long-term Memory CD8^+^ T Cell Responses

Mice were immunized with RAS (70K-20K-10K-10K) intravenously (i.v.) at biweekly interval as shown earlier (19) and challenged on day 60 with 10K Inf. Spz (Inf. Spz group) or RAS (RAS group). On day 232, both the groups were challenged with Inf. Spz to recall the LS-specific memory T cell responses. On day 3 post-challenge, CD8^+^ T cells in the liver were characterized (Figure S1 in Supplementary Material).

#### Immune Status of DCs

Mice were inoculated i.v. with 10K, 50K RAS or 10K Inf. Spz. Control mice (sham group) were injected with purified material isolated from salivary glands of uninfected mosquitoes called as sham dissection, representing equivalent number of infected mosquitoes. Mononuclear cells (MNCs) were harvested from the liver, spleen, and liver-draining LNs (28) and DCs were characterized on 72 h postinoculation.

In parallel experiment, we looked for the accumulation and the activation status of the DCs in immune mice. For this, mice were primed with 50K RAS through i.v. and were subsequently challenged with 10K Inf. Spz or RAS on day 15. DCs were characterized, as explained above, 72 h postinoculation or challenge.

#### Induction of CD4^+^ T Cells

Mice were primed with 50K RAS through i.v. Some of the mice were challenged with 10K Inf. Spz or RAS on day 15 post-priming. CD4^+^ T cells were characterized from the liver on day 3 post-challenge.

### Isolation of MNCs

Mice were anesthetized by ketamine (80 mg/kg body weight) and xylazine (6 mg/kg body weight). Upon dissecting the mouse, liver was perfused by flowing 10–15 ml PBS at the rate of 10 ml/min and inferior vena cava was cut for blood out flow. Gall bladder was removed and liver was pass through a 70-µM cell strainer (Himedia, India) with the help of 10 ml syringe plunger. Cell suspension was washed, resuspended in the 33% Percoll (GE healthcare) in PBS, and centrifuged for 20 min at 800 × *g*. Cell suspensions were prepared by following the established protocol and RBCs were lysed with ACK RBC lysis solution, washed twice with PBS. The cell counting was done on Neubauer Chamber.

### Flow Cytometry Analysis

#### Characterization of CD8^+^ or CD4^+^ T Cells

1.5–2.0 million cells were suspended in FACs buffer. PBS supplemented with 1–2% fetal calf serum for cell surface staining and intracellular cytokine staining (ICS). Seven-color staining of intrahepatic mononuclear cells were performed by using monoclonal antibodies. The following mouse monoclonal antibodies were used in different combinations as per the requirement of an experiment. PerCP-conjugated anti-CD3 (145-2C11 clone), APC-H7-conjugated anti-CD8α (53-6.7 clone), PE-Cy7-conjugated anti-CD44 (IM7 clone), V450-conjugated anti-CD62L (MEL-14 clone), FITC-conjugated anti-CD107a (1D4B clone), and APC-conjugated anti-IFN-γ (XMG1.2 clone), V500-conjugated anti-CD4 (RM4-5 clone), PerCP-conjugated anti-CD4 (RM4-5 clone), PE/Cy7 anti-CD154 (CD40L) (MR1 clone), and V500-conjugated anti-CD44 (IM7 clone) (BD Bioscience or BioLegend), respectively. For cell surface and ICS, 2 × 10^6^ cells were resuspended in FACS buffer and incubated for 20 min at 4°C with monoclonal antibodies for cell surface markers. Cells were fixed and permeabilized with intracellular (IC) fixation buffer and washed with Perm wash buffers (eBiosciences). Cells were again incubated with anti-IFN-γ monoclonal antibody for 30 min at RT for ICS. Simultaneously, a control sample was kept in which cells were incubated with Isotype control (Rat IgG1, κ) instead of anti-IFN-γ antibody. After washing with Perm wash buffer and FACs buffer, cells were finally resuspended in 1% para-formaldehyde for cell acquisition by flow cytometer.

#### Phenotypic Characterization of DCs

1.5–2.0 million cells were suspended in FACs buffer. PBS supplemented with 1–2% fetal calf serum for cell surface staining. The following mouse monoclonal antibodies were used in each experiment: PE Anti-CD11c (Clone: N418), FITC anti-MHCII (Clone: M5/114.15.2), PerCP anti-CD86 (Clone: GL-1), PE Cy-7 anti-CD40 (Clone: 3/23), APC anti-F4/80 (Clone: BM8) (BioLegend), APC-H7 anti-CD8α (Clone: 53-6.7), and V450 anit-CD80 (Clone: 16-10A1) (BD Bioscience), respectively. Isotype controls were used for each cell surface marker.

Cells were acquired on FACS Aria-III equipped with three lasers flow machine at Central Facility, MSU, Vadodara or Beckman Coulter CytoFLEX platform. The cells were gated according to forward- and side-scatter to eliminate the dead cells and debris for analysis. Moreover, we have discriminated doublets using the FSC-H vs. FSC-A strategy, and flow cytometer raw data were analyzed by using FlowJo_v10 software (USA).

### Liver Microenvironment in Response to Sporozoite Inoculation

Three groups of five mice each were inoculated i.v. with 50K RAS, 10K Inf. Spz, or sham dissected salivary gland material from uninfected mosquitoes with similar dilution. After 72 h postinoculation, mice were euthanized and livers were perfused using PBS and were immediately submerged in RNA later solution to protect degradation of RNA.

### RNA Sequencing, Assembly, and Annotation

Transcriptome sequencing was carried out on an Illumina NextSeq platform that generated approximately 75-bp paired-end (PE) raw reads. The raw data were filtered using Trimmomatic v0.30 software (29). After removing adaptor sequences, ambiguous “N” nucleotides (with the ratio of “N” greater than 5%) and low-quality sequences (with quality score less than 10), the remaining high-quality reads were aligned and pull together using TopHat and Bowtie software (30) as described for aligning the high-quality reads with a *Mus musculus* reference genome. Raw sequencing data of all the samples is available through the NCBI Sequence Read Archive accession: SRP129582.^1^ For homology annotation, non-redundant sequences were subjected to public databases, including NCBI non-redundant protein and non-redundant nucleotide, Swiss-Prot,^2^ gene ontology (GO) by panther classification system,^3^ and the pathway analysis by Kyoto Encyclopedia of Genes and Genomes (KEGG).^4^

### Analysis of Differentially Expressed Genes

To analyze initial host response against RAS and Inf. Spz, differentially expressed genes in *M. musculus*, the number of reads for each of the sequenced reads from the two samples was converted to fragments per kilo base of transcript per million mapped reads (FPKM) (31). The criteria of each gene having a read depth of minimum 5 reads was also applied for finding differentially expressed genes. Apart from the FPKM value, *p*-value and a fold change value were also calculated for sham and Inf. Spz inoculated groups. Subsequently, pathway enrichment analysis was done by KEGG pathway analyser server,^5^ which is widely used in functional annotation and enrichment analysis. The ontology covers three domains namely Cellular Component, Molecular Function and Biological Process. The GO annotations in panther are either obtained from public databases (panther classification system or KEGG pathway analyser server) or produced by computational predictions.

### Gene Expression Study and Validation

Genes identified in the transcriptome sequencing analysis were validated by quantitative real-time PCR (RT-qPCR). The primers (Table S1 in Supplementary Material) were taken from https://pga.mgh.harvard.edu/primerbank/. The prepared total RNA used in quantitative real-time PCR analysis was isolated from the same sample as that used for RNA sequencing. The quantitative real-time PCR was performed on the ThermoScientific Quant studio 3 real-time PCR system using 2 × SYBR green real-time PCR mix (Clontech, Takara). The PCR amplification was performed in triplicate, using the following cycling parameters: 94°C for 2 min, followed by 40 cycles of 10 s at 94°C, 10 s at 59°C, and 34 s at 72°C. All samples were analyzed in triplicate and the expression of target genes was calculated as relative fold values using the 2−ΔΔCT method.

### Statistical Analysis

The average values ± SEM are presented in each graph and table. Statistical analysis was performed using GraphPad Prism software version 7.0 for Windows (GraphPad Software). *p* values were calculated by using non-parametric Mann–Whitney *U*-test. *p* Value ≤0.05 was considered statistically significant.

## Results

### Infectious Sporozoite Challenge Brings Qualitative Changes in RAS-Induced Memory CD8^+^ T Cells

**Figure 1:**
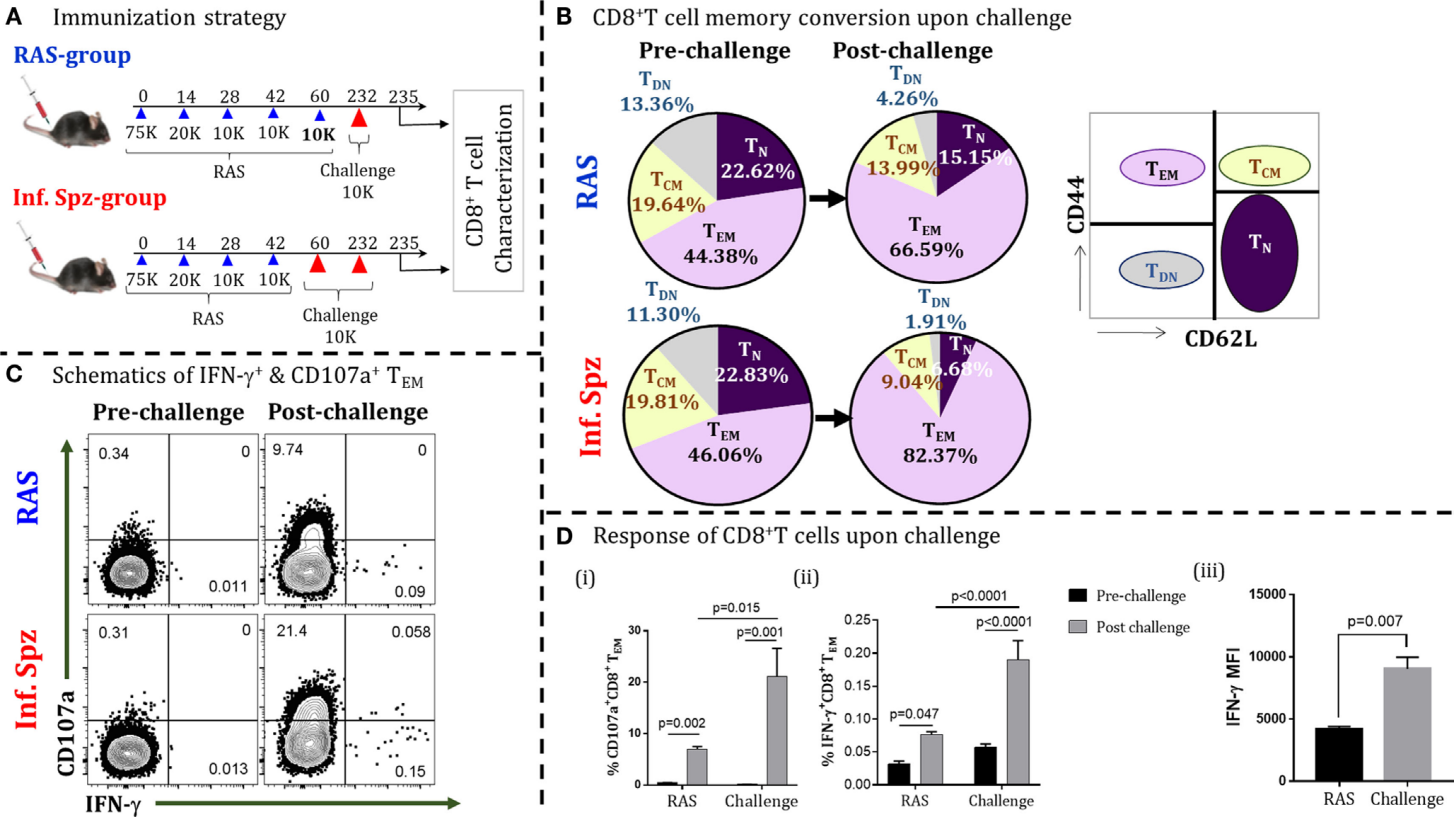
Infectious sporozoites (Inf. Spz) challenge induces qualitatively different liver-stage specific CD8^+^ T cells in radiation-attenuated sporozoites (RAS) immunized mice. **(A)** Immunization strategy. C57BL/6 mice were immunized with RAS (70K-20K-10K-10K) intravenously at biweekly interval. Some of the RAS immune mice were challenged with 10K Inf. Spz on 2–3 weeks post last-boost immunization (Inf. Spz group) while others were challenged with 10K RAS in place of Inf. Spz (RAS group). Mice from both groups were challenged on day 232 for the reactivation of liver-stage specific memory CD8^+^ T cells. The T cells were characterized on day 3 post-challenge (Figure S1 in Supplementary Material). **(B)** Pie chart analysis of the CD8^+^ T-cell subsets in RAS- and Inf. Spz groups pre- and day 3 post-challenge: naïve (T_N_) (CD44^−^CD62L^+^), central memory (T_CM_) (CD44^+^CD62L^+^), effector memory (T_EM_) (CD44^+^CD62L^−^), and double-negative (T_DN_) (CD44^−^CD62L^−^) phenotypes. **(C)** Representing dot plots of IFN-γ^+^ and CD107a^+^ CD8^+^ T_E/EM_ cells of RAS or Inf. Spz group **(D)** (i) % CD107a^+^, (ii) IFN-γ^+^ CD8^+^ T_E/EM_ cells, and (iii) The expression levels of IFN-γ in CD8^+^ T_E/EM_ cells measured in terms of mean fluorescent intensity (MFI) day 3 post-challenge. *N* = 6 mice per group. Data are the mean ± SEM. The data were analyzed with non-parametric Mann–Whitney *U*-test. *p* < 0.05 is considered as a significant. Data shown represent one of two independent experiments.

Induction of liver-stage immunity to *Plasmodium* infection in animals and humans requires multiple immunizations of RAS (4, 6, 32). Whole sporozoites vaccination (WSV) has been shown to render sterile and protracted protection against multiple challenges (4). In B6 mouse model, we found that Pb RAS-induced sterile protection is maintained for 6 months (11). Interestingly, the intermittent Inf. Spz challenge following last-boost immunization was observed to prolong the sterile protection up to 18 months (11). While the loss of protection within 6 months was associated with the sharp decline of CD8^+^ T cells in liver, the intermittent challenge had rescued the CD8^+^ T cells from attrition. These observations were based on phenotypic and functional characterization of the memory CD8^+^ T cells without knowing their antigen specificities. Here, we wanted to understand the qualitative changes in LS-specific CD8^+^ T cell response upon challenge that might be responsible for ensuing longer-lived protection. We immunized B6 mice with four doses of Pb RAS (75K-20K-10K-10K) with an interval of 2 weeks apart as described previously (19). On day 60, some of the immunized mice challenged with 10K Inf. Spz (now onward termed as Inf. Spz group), while others were challenged with 10K RAS (now onward termed as RAS group). Since RAS immunized mice were protected for 6 months, we sought to determine the reactivation potential of CD8^+^ T cells after 6 months in order to find out the differences between RAS and Inf. Spz groups. On day 232, both the groups were challenged with 10K Inf. Spz as shown in Figure 1A and LS-specific memory CD8^+^ T cells were characterized on day 3 post-challenge.

Generally, the antigen-specific T cell responses are measured in the form of IFN-γ expression after *in vitro* stimulation with specific peptide or protein antigens. Since majority of the *Plasmodium* LS-specific antigens are yet to be discovered, measuring the LS-specific CD8^+^ T cell response through *in vitro* stimulation by limited available antigens we may bias our observation and the same might not truly reflect the nature of sterile protection. Moreover, inducing the LS-specific sterilizing immunity requires the hepatocytes to be invaded by the sporozoites for the expression of the LS antigens to effectively activate the CD8^+^ T cells. To reactivate the memory CD8^+^ T cells to any possible LS antigens, we followed the *in vivo* T cell reactivation approach (33). For this, CD8^+^ T cells generated following RAS immunization were reactivated by sporozoites challenge and IFN-γ response was measured by the *in vivo* ICS (33) along with cytotoxic response (CD107a^+^).

Upon challenging the mice with Inf. Spz for reactivating the T cells, we found significant elevation of effector/effector memory CD8^+^ T cells (T_E/EM_; CD44^hi^CD62L^lo^) in their liver (post-challenge; day 235) compared to the same in mice without challenge (pre-challenge; day 232), suggesting that T cells respond to antigen challenge. Interestingly, the elevation of T_E/EM_ cells was found higher in the Inf. Spz group (46.06–82.37%) compared to RAS group (44.38–66.59%) (Figure 1B). Previously, we have shown that LS-specific CD8^+^ T_CM_ cells are critical for protracted protective immunity (7). The reduction in CD8^+^ T_CM_ cells associated with the elevation of CD8^+^ T_E/EM_ cells is used as a measure of the memory T cells being reactivated to antigenic challenge, thus converting T_CM_ to T_E/EM_. In this study, we found the reduction of central memory CD8^+^ T cells (T_CM_; CD44^hi^CD62L^hi^) upon reactivation on day 232 post last immunization. While the RAS group showed about 5% reduction (i.e., 19.64–13.99%), the Inf. Spz group demonstrated twofold higher reduction in T_CM_ population 19.81–9.04% (Figure 1B). The above results depict that either the reactivation potential of memory T cells is higher or the frequencies of memory T cells maintained in the Inf. Spz group compared to RAS group as reflected in the conversion of T_CM_ to T_E/EM_. To further understand the qualitative changes in the effector function of the responding T cells, we measured the IC IFN-γ production and cytotoxic activity (CD107a^+^) of the CD8^+^ T_E/EM_ cells (Figure S1 in Supplementary Material). The results suggest the significant activation of cytotoxic (CD107a^+^) and IFN-γ-producing CD8^+^ T cells in both the groups after challenge (Figure 1C). The percentage of IFN-γ^+^ cells is lower in general that we observed consistently in our experiments. However, we found threefold higher accumulation of cytotoxic T cells (*p* < 0.05) in the liver of Inf. Spz group compared to RAS (Figure 1Di). Similarly, the frequencies of IFN-γ producing CD8^+^ T cells was 2.5-fold (*p* < 0.0001) higher in the Inf. Spz group (Figure 1Dii). It is noteworthy that we found not just quantitative, but also the qualitative difference in terms of IFN-γ production per cell. The intermittent challenge helped generate high IFN-γ producing T cells compared as presented in the form of mean fluorescent intensity (MFI) (Figure 1Diii). The reactivation of high frequencies of cytotoxic (CD107a^+^) and IFN-γ-producing CD8^+^ T cells in Inf. Spz group correlates with the higher percentage CD8^+^ T_E/EM_ cells. Collectively, these results suggest that intermittent challenge with Inf. Spz following RAS vaccination generates LS-specific CD8^+^ T cells, which are qualitatively better. It is possible that the generation of qualitatively different CD8^+^ T cells could be the result of differential innate immune response between RAS and Inf. Spz groups.

### Infectious Status of Sporozoites Induces Interferon Signaling *via* Toll-Like Receptor (TLR) Modulation

**Figure 2:**
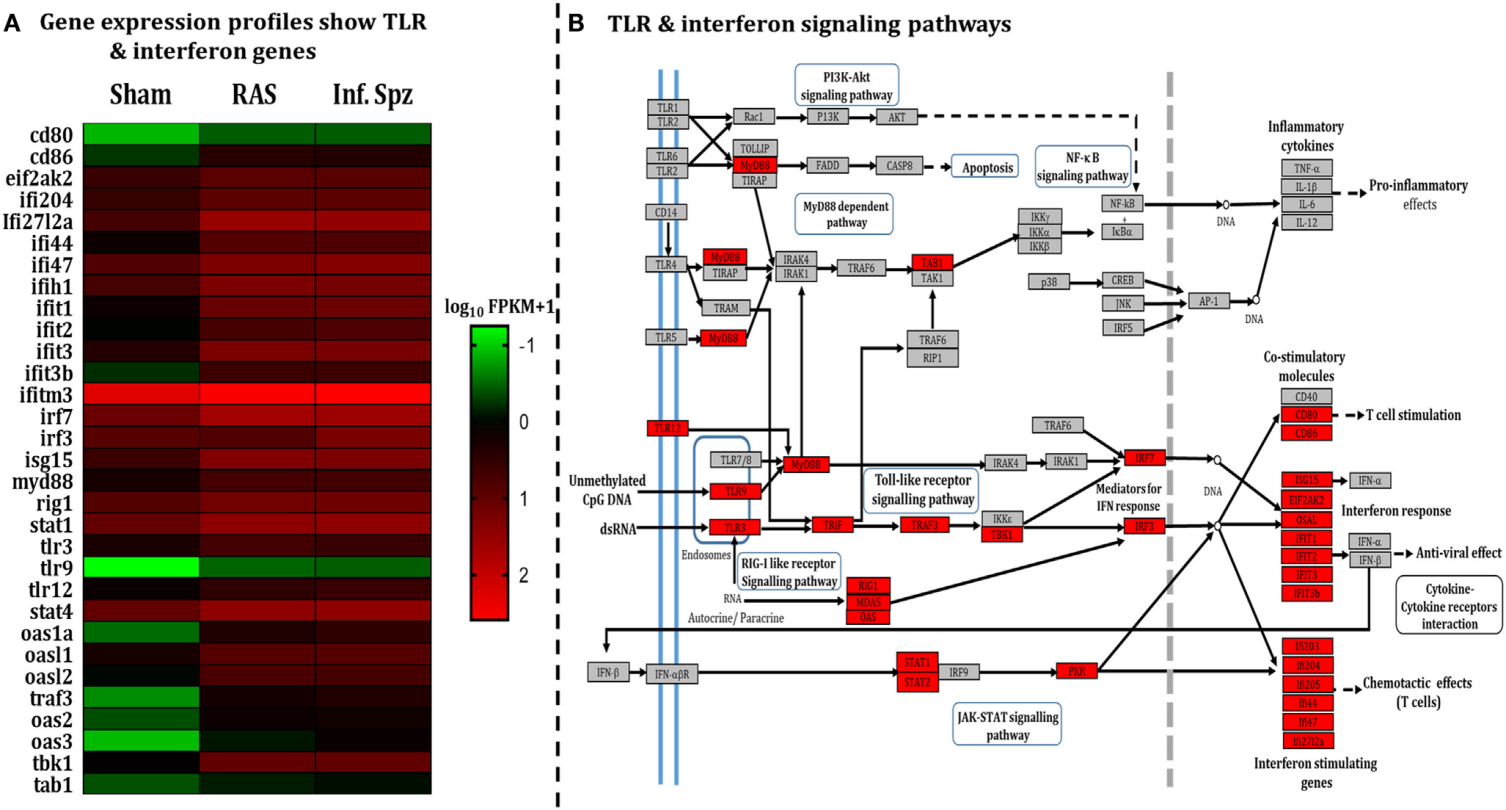
Assessment of liver microenvironment through whole-genome RNA Seq. **(A)** Heat map showing differential gene expression in mouse liver post 72 h following 50K radiation-attenuated sporozoites (RAS) or 10K infectious sporozoites (Inf. Spz) inoculation. **(B)** Enriched pathway in Kyoto Encyclopedia of Genes and Genomes (KEGG) database: significantly differentially expressed genes identified by KEGG as involved in toll-like receptor signaling and interferon signaling. Red, significantly increased expression (fold change > 1); grey, unchanged expression.

The pattern of LS-specific CD8^+^ T cell activation in response to intermittent sporozoites challenge prompted us to look for the differential microenvironment created in the liver during the challenge (34–37) that might modulate the antigen-presenting cells (APCs). Hence, we did a comparative study involving RNA-seq liver transcriptome analysis for which mice were inoculated with RAS, Inf. Spz, or sham control. Results revealed statistically significant differences in the expression of 590 transcripts (*p* < 0.05), out of which 430 genes were upregulated in case of Inf. Spz as compared to sham control mice. Of those 430 genes, 363 transcripts were at least onefold higher (*p* < 0.05). Subsequently, we found that 105 genes out of 363 genes involved in immune response (Figure S2A and Table S2 in Supplementary Material). Further, 31 genes out of 105 immune response-related genes found for their involvement in the TLR signaling pathway (Figure 2A; Table S2 in Supplementary Material) that induce interferon response associated with activation of APCs (38). By quantitative RT-PCR, we validated 9 upregulated genes (Figure S2B in Supplementary Material) that are found to be involved in TLR signaling as analyzed by KEGG pathway database server (Figure 2B).

### Infectious Sporozoite Inoculation Increases the Accumulation of CD8α^+^ DCs in Liver

**Figure 3:**
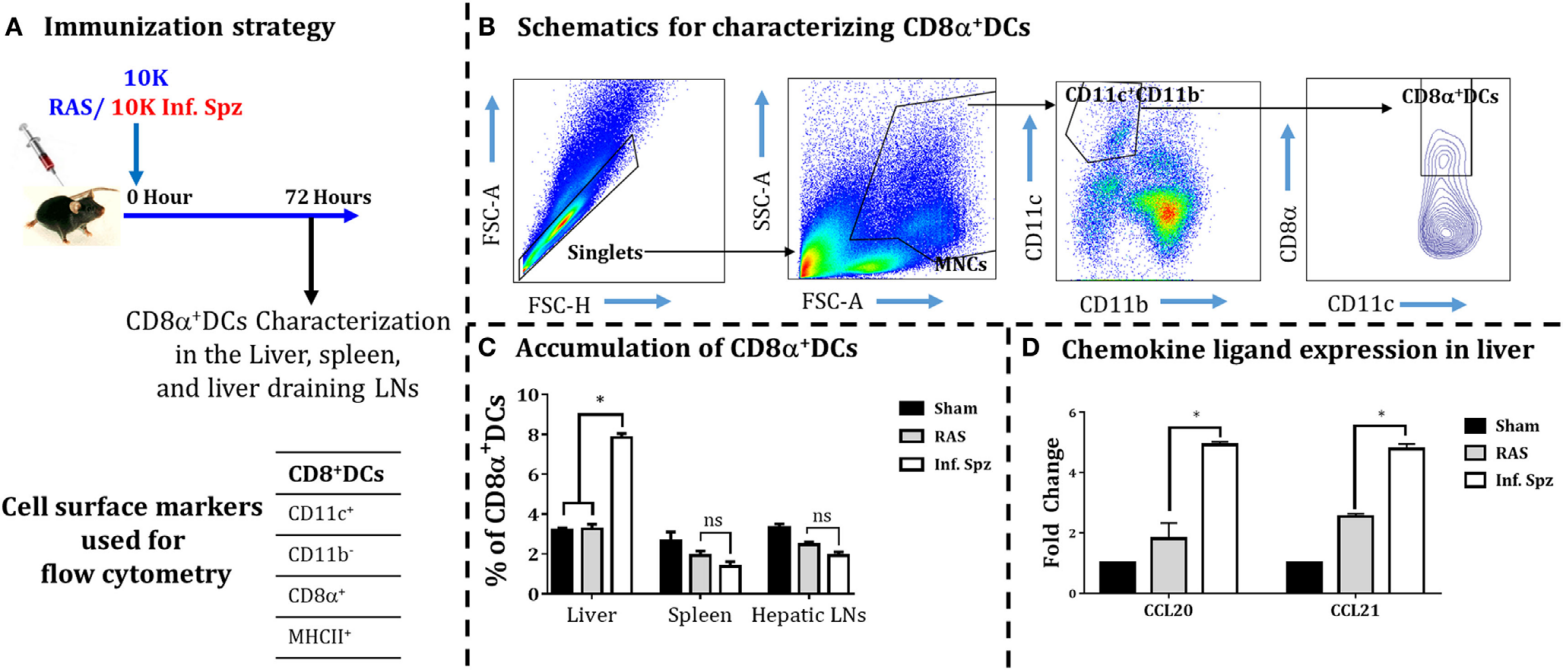
Accumulation of dendritic cells (DCs) in different organs [liver, spleen, and liver-draining lymph nodes (LNs)] in response to radiation-attenuated sporozoites (RAS) or infectious sporozoites (Inf. Spz) inoculation. **(A)** C57BL/6 mice were inoculated with 10K RAS or Inf. Spz and mice sacrificed 72 h postinoculation. **(B)** Gating strategy for characterizing DCs, used to define CD11c^+^CD11b^−^CD8α^+^ DCs within the mononuclear cells (MNCs). Murine DC subsets were differentiated *via* expression of CD8α on CD11c^+^CD11b^−^ cells from liver, spleen, and liver-draining LN. The FACS plot is a representation from the liver. **(C)** Accumulation of CD8α^+^ DCs in liver and lymphoid organs. The graphs present the percent cells out of total MNCs. Each bar shown in graph is mean of four mice per group. *p < 0.05, relative to corresponding RAS inoculated group. **(D)** Measurement of the expression of chemokine ligands (CCL20 and CCL21) in RAS or Inf. Spz inoculated mice through qPCR. The expression of β-actin gene in individual sample was used as an internal control for each mouse. Data are expressed as the fold induction of the gene of interest in the different activation conditions compared with sham control group. Data are the mean ± SEM. The data were analyzed with non-parametric Mann–Whitney *U*-test. *p* < 0.05 is considered as a significant. *N* = 4 mice per group. Data shown represent one of two independent experiments.

Earlier it was reported in mouse models of various viral infections as well as IC parasites that the DCs accumulate at the site of infection (16, 39–41). Since the activation of malaria LS-specific CD8^+^ T cells is also shown to be critically dependent on the CD8α^+^ DCs (16, 25, 26, 42), we wanted to determine whether the inoculation of Inf. Spz would promote higher accumulation of DCs in the liver compared to RAS inoculation. Mice were given inoculums of 10K RAS or Inf. Spz and at 72 h postinoculation the DCs characterized as CD11c^+^CD11b^−^ population were further analyzed for CD8α^+^ subset of DCs in the liver, spleen, and hepatic LNs (Figure 3) as shown in earlier findings (24, 43, 44). We found that 7.8 ± 0.21% (*p* < 0.05) of DCs in the liver of Inf. Spz inoculated mice expressed CD8α, whereas only 3.2 ± 0.24% DCs in the liver of RAS mice expressed CD8α (Figure 3C). The status of DCs in spleen or LNs was not significantly different between Inf. Spz or RAS inoculated mice. Nevertheless, the presence of relatively high frequencies of CD8α^+^ DCs in Inf. Spz inoculated mice suggests that exposure to Inf. Spz enhances the accumulation of CD8α^+^ DCs in the liver compared to RAS. We suspect that accumulation of CD8α^+^ DCs in the liver could be the outcome of enhanced migration of these cells which is critically dependent on the availability of specific chemokines required for the migration of DCs to the site of infection (45). Therefore, we next determined whether infectious status of sporozoites modulates the inflammatory milieu of liver in terms of the expression of chemokine ligands, CCL20, and CCL21. We performed real-time RT-PCR from the liver of sham, RAS, or Inf. Spz inoculated mice. We observed about twofold increased expression of CCL20 (*p* < 0.05) and CCL21 (*p* < 0.05) in response to Inf. Spz inoculation as compared to that of RAS inoculation (Figure 3D). Collectively, these results suggest that the Inf. Spz inoculation promotes the greater accumulation of CD8α^+^ DCs to the site of infection probably by modulating the liver microenvironment.

### The Infectious Sporozoite Modulates Greater Expression of MHCII and Costimulatory Molecules on CD8α^+^ DCs

**Figure 4:**
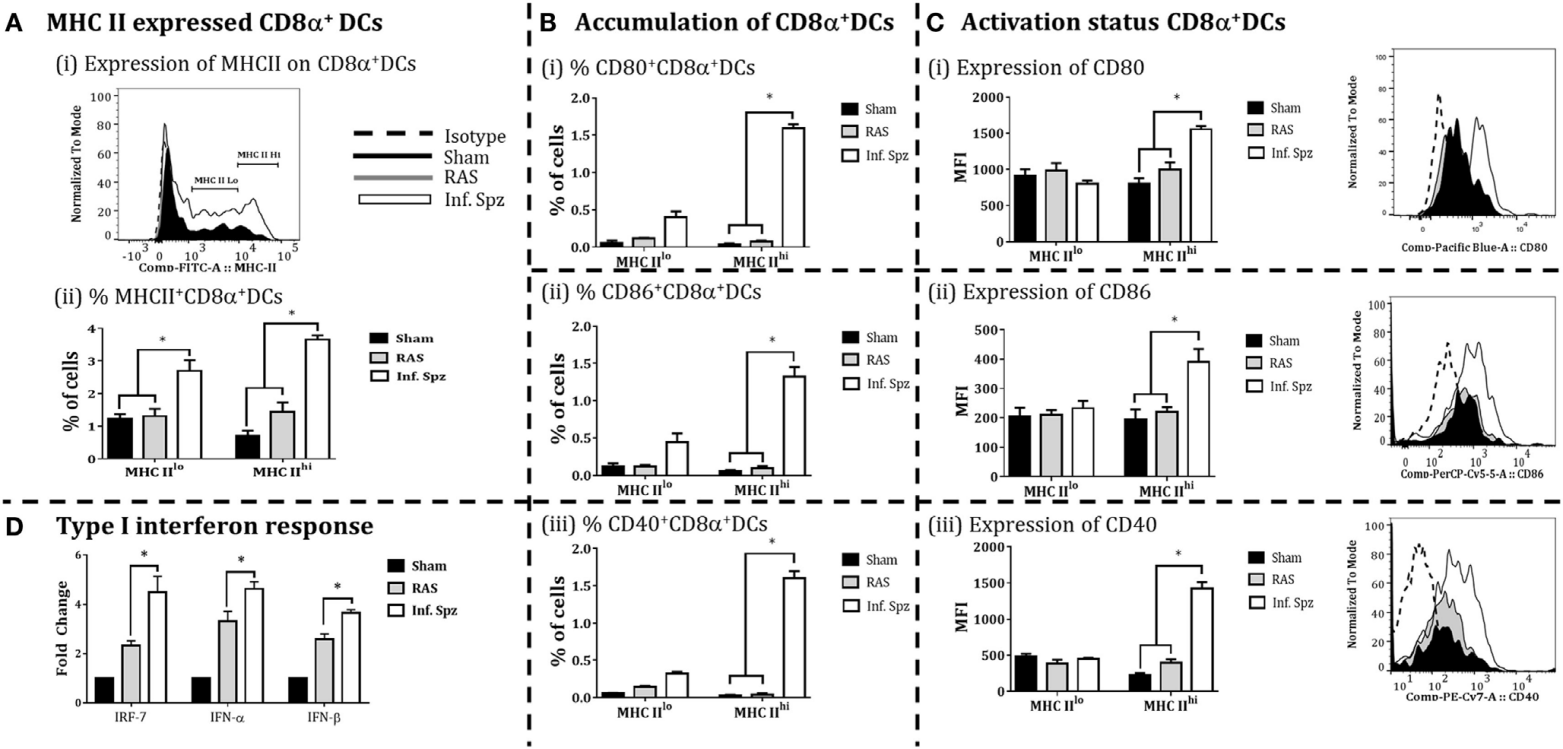
CD8α^+^ dendritic cells (DCs) expressed the MHC class II (I-A^b^) and costimulatory molecules; CD80, CD86, and CD40 in the liver. **(A)** Percent CD8α^+^ DCs with MHC-II high (MHCII^hi^) and MHC-II low (MHCII^lo^) accumulated in the liver and histogram showing MHC-II expression on CD8α^+^ DCs. **(B)** Accumulation of CD8α^+^ DCs with the expression of (i) CD80, (ii) CD86, and (iii) CD40. The graphs present the percent cells out of total mononuclear cells. **(C)** The expression of (i) CD80, (ii) CD86, and (iii) CD40 measured as the mean fluorescent intensity (MFI) on CD8α^+^ DCs (*left panels*) in the liver. The *right panels* show histogram for the expression of CD80, CD86, and CD40 on MHC-II^hi^ CD8α^+^ DCs in the liver. **(D)** Measurement of the interferon response (IRF-7, IFN-α, and IFN-β) in radiation-attenuated sporozoites (RAS) or infectious sporozoites (Inf. Spz) inoculated mice through qPCR. The expression of β-actin gene in individual sample was used as an internal control for each mouse. Data are expressed as the fold induction of the gene of interest in the different activation conditions compared with sham control group. Data are the mean ± SEM. The data were analyzed with non-parametric Mann–Whitney *U*-test. *p* < 0.05 is considered as a significant. Data shown represent one of two independent experiments.

The accumulation of high frequency CD8α^+^ DCs in the liver might not guarantee better CD8^+^ T cell responses as the effective adaptive immunity is critically dependent on the immune status of APCs (46). In our study, we observed the differences in the frequencies of CD8α^+^ DCs; next we determined the activation status of these DCs in the liver by measuring the expression of MHC-II molecules, costimulatory molecules CD80 (B7.1), CD86 (B7.2), and CD40 on the CD8α^+^ DCs by flow cytometry to understand whether Inf. Spz inoculation modulates the activation of CD8α^+^ DCs found in the liver. First, we segregated the CD8α^+^ DCs, for their MHC-II upregulation, into MHC-II^lo^ and MHC-II^hi^ populations (Figure 4Ai). We found that Inf. Spz significantly increased frequencies of MHC-II^hi^ (3.65 ± 0.13%, *p* < 0.05) as well as MHC-II^lo^ (2.7 ± 0.33%, *p* < 0.05) CD8α^+^ DCs in the liver compared to that of RAS (1.4 ± 0.29%, 1.3 ± 0.22%) and sham control (0.7 ± 0.16%, 1.2 ± 0.21%) mice (Figure 4Aii).

As DCs having higher MHC-II are considered matured APCs, we focused on MHC-II^hi^ populations for their expression of costimulatory molecules B7.1 (CD80) and B7.2 (CD86). We found that 35–50% of MHCII^hi^ CD8α^+^ DCs in the liver of Inf. Spz mice expressed CD80 and CD86 (Figure 4Bi,ii), whereas significantly low% of the same population expressed the costimulatory molecules in RAS (~10%, *p* < 0.05) and sham control (5%, *p* < 0.05) mice (Figure 4Bi,ii). These observations became more interesting when we individually determined the levels of CD80 or CD86 expression on MHCII^hi^ populations. While the levels of CD80 (MFI = 1,554 ± 46.4) or CD86 (MFI = 390 ± 43.9) expression on MHCII^hi^CD8α^+^ DCs was higher in Inf. Spz inoculated mice compared to RAS CD80 (MFI = 805 ± 73) or CD86 (MFI = 194 ± 34.6), the same was not significantly different in RAS inoculated mice CD80 (MFI = 994 ± 103) or CD86 (MFI = 221 ± 15.9) (Figures 4B,C).

The CD40 expression on DCs is shown to be associated with enhanced CD8^+^ T cell mediated protective response against *Plasmodium* (26). Hence, we measured expression of CD40 on the matured liver CD8α^+^ DCs in response to RAS or Inf. Spz inoculation. We found higher % of MHCII^hi^CD8α^+^ DCs express CD40 molecules in response to Inf. Spz inoculation (1.60 ± 0.09%; *p* < 0.05). The CD40 expression levels were estimated highest on the above population in Inf. Spz inoculated mice (*p* < 0.05) (Figure 4Biii) followed by RAS and sham control (MFI: 1,421 ± 91.76 in Inf. Spz, 397 ± 49.25 in RAS, 230 ± 24.6 in sham) (Figure 4Ciii).

The immune status of CD8α^+^ DCs is highly influenced by the cytokine microenvironment. Particularly, type I interferons play critical role to fully activate the DCs. We measured the expression of type I interferons (IFN-α, β) and the transcription factor IRF-7 (interferon regulatory factor-7) in the liver to determine the differential responses in RAS vs. Inf. Spz inoculated mice. The expression of cytokines at mRNA level was assessed by quantitative real-time PCR in liver after 72 h postinoculation. We observed significantly higher expression of IRF-7 (*p* < 0.05), IFN-α (*p* < 0.05) and IFN-β in the liver upon Inf. Spz than RAS inoculation (Figure 4D). These results suggest that the activation status of liver CD8α^+^ DCs in RAS or Inf. Spz inoculated mice could be the outcome of the differential expression of type I interferons.

### Differential Dose of RAS Correlates with the Accumulation and Activation of CD8α^+^ DCs in the Liver

**Figure 5:**
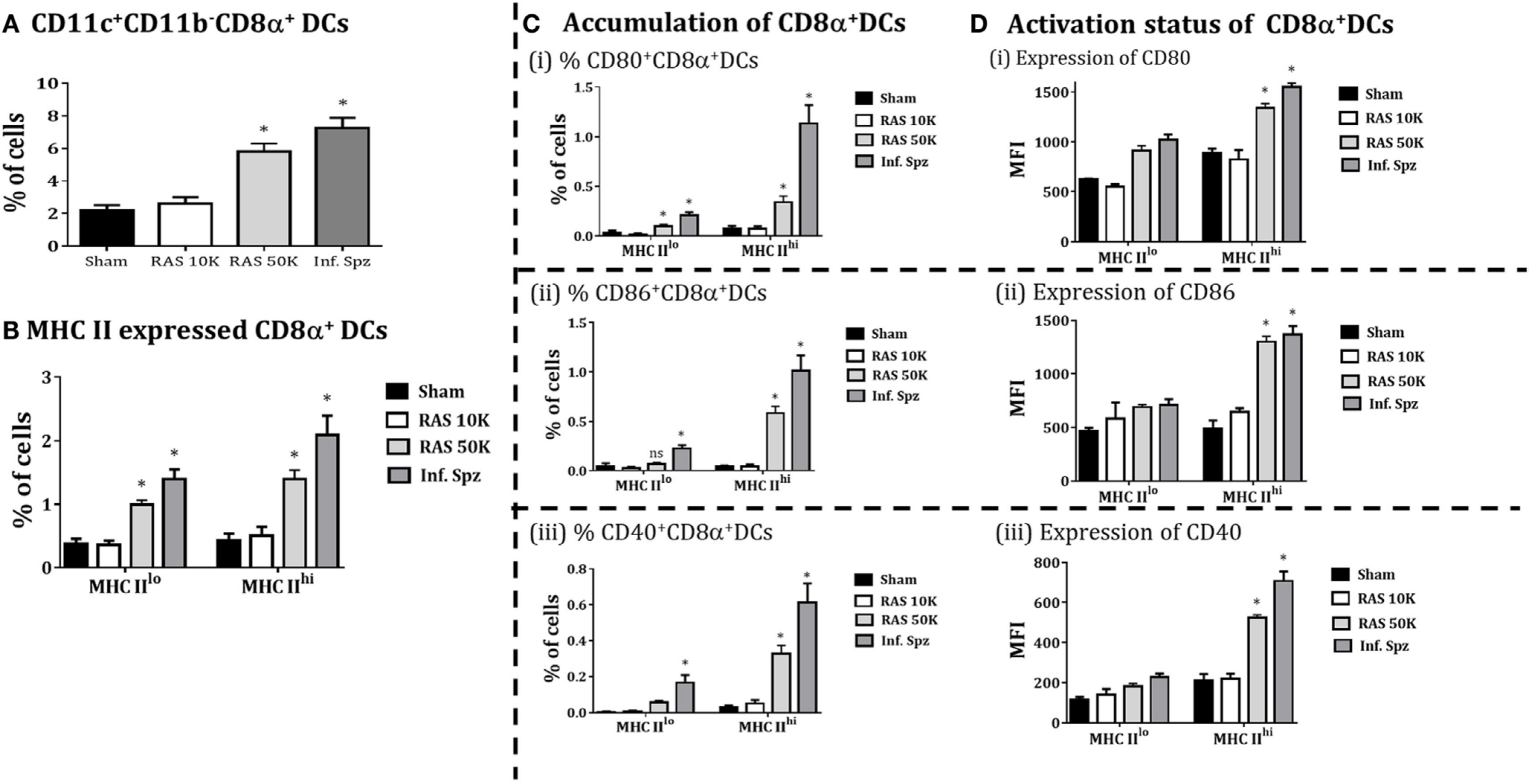
CD8α^+^ dendritic cells (DCs) in response to differential dose of radiation-attenuated sporozoites (RAS) (10K or 50K). **(A)** Accumulation of CD8α^+^ DCs is significantly increases with dose 50K RAS as compared to 10 K RAS as shown in the figure, **(B)** CD8α^+^ DCs subdivided in MHC-II high (MHCII^hi^) and MHC-II low (MHCII^lo^) DCs, **(C)** accumulation of CD8α^+^ DCs with expression of CD80, CD86, and CD40, **(D)** activation status of CD8α^+^ DCs with the expression of MHCII (high or low), shown expression of CD80 and CD86 measured as the mean fluorescent intensity (MFI) in CD8α^+^ DCs in the liver. Percent cell shown is the percent out of total mononuclear cells. *N* = 4 mice per group. Data are the mean ± SEM. The data were analyzed with non-parametric Mann–Whitney *U*-test. *p* < 0.05 is considered as a significant. Data shown represent one of two independent experiments.

Because Inf. Spz have ability to complete the liver-stage developmental cycle, the enhanced CD8^+^ T cell responses by Inf. Spz challenge may be associated with the development of parasite in liver. Unlike Inf. Spz, RAS undergoes aborted development in the liver which might limit the expression profiles of pathogen associated molecules in relation to inflammatory responses. Therefore, we wanted to dissect whether increased dose of RAS inoculation could surpass the accumulation and activation levels of CD8α^+^ DCs in the liver that was found with 10K RAS or inf. Spz inoculation. We inoculated mice with 10K or 50K RAS and evaluated the status of CD8α^+^ DCs in the liver 72 h postinoculation by comparing the same with mice inoculated with 10K Inf. Spz. We found that the accumulation of CD8α^+^ DCs in the liver of mice inoculated with 50K RAS doubled compared to that of 10K RAS, which is still lower than 10K Inf. Spz inoculation (Figure 5A). Upon determining the modulatory role of sporozoites inoculum on CD8α^+^DCs in liver, we found that 50K RAS significantly enhanced the upregulation of MHC-II (Figure 5B) as well as expression of CD80 (MFI = 1,340 ± 42.4, *p* < 0.05), CD86 (MFI = 1,301 ± 49.9, *p* < 0.05), and CD40 (MFI = 533 ± 15.4, *p* < 0.05) compared to 10K RAS (Figure 5D). Interestingly, we found that the % of MHCII^hi^CD8α^+^ DC expressing CD80, CD86 or CD40 in the liver of 50K RAS inoculated mice are still appreciably lower than that of Inf. Spz; CD80 (0.338 ± 0.06%, 50K RAS vs. 1.13 ± 0.18%, Inf. Spz *p* < 0.05), CD86 (0.58 ± 0.06%, 50K RAS vs. 1.01 ± 0.16%, Inf. Spz *p* < 0.05), and CD40 (0.32 ± 0.046%, 50K RAS vs. 0.613 ± 0.10%, Inf. Spz *p* < 0.05) (Figure 5C).

### Infectious Sporozoite Challenge following RAS Priming Promotes the Accumulation As Well As Activation of CD8α DCs in Liver

**Figure 6:**
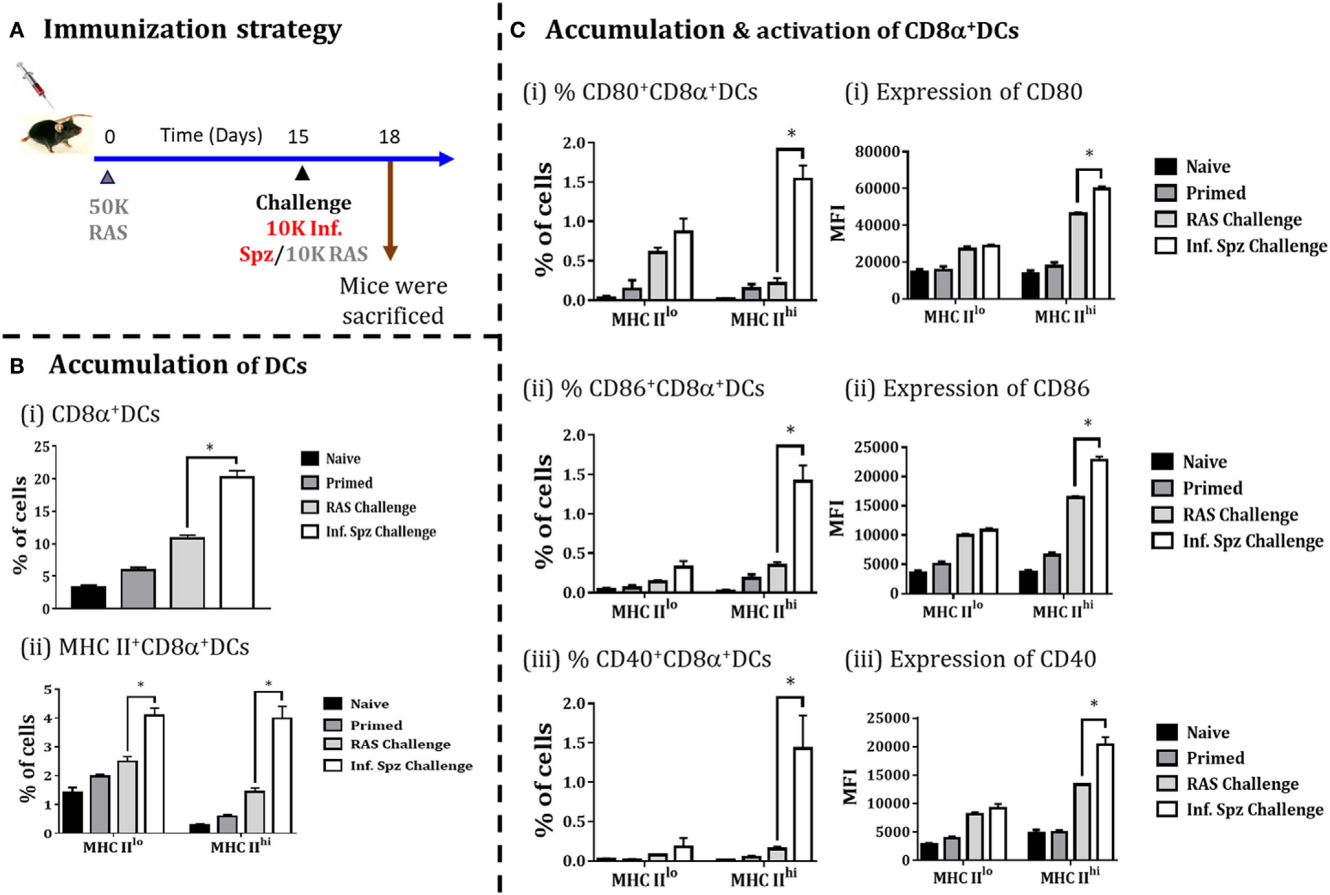
Accumulation of CD8α^+^ dendritic cells (DCs) in the liver in response to challenge with infectious or radiation-attenuated sporozoites (RAS) (10K each) after priming. **(A)** Immunization strategy. Mice were primed with 50K RAS, on day 15 one group of mice were challenged with 10K infectious sporozoites (Inf. Spz) and another group with 10K RAS. Mice were sacrificed 72 h post-challenge, **(B)** Accumulation of CD8α^+^ DCs in the liver of mice post-challenge. **(C)** Panel showing accumulation and activation status of CD8α^+^ DCs with the expression of various costimulatory molecules (CD80, CD86, and CD40), (i) CD80, (ii) CD86, and (iii) CD40. Each bar shown in graph is mean of four individual mice per group. Percent cell shown is the percent out of total mononuclear cells or lymphocytes. *N* = 4 mice per group. Data are the mean ± SEM. The data were analyzed with non-parametric Mann–Whitney *U*-test. *p* < 0.05 is considered as a significant. Data shown represent one of two independent experiments.

Whole sporozoites vaccination approach requires a multiple immunizations to elicit the protective threshold frequencies of LS-specific CD8^+^ T cells, required to confer sterile protection against *Plasmodia* infection (19). As explained earlier, Inf. Spz challenge following multiple RAS immunizations plays a critical role in enhancing the longevity of the sterile protection by maintaining the frequencies of LS-specific CD8^+^ T cells (Figure 1). Therefore, we asked whether Inf. Spz challenge modulates the accumulation of CD8α^+^ DCs in RAS inoculated mice. The mice were primed with RAS (50K) i.v. and on day 15 post-priming, one of the groups of mice were challenged with 10K Inf. Spz, whereas another group received 10K RAS i.v. After 72 h, the mice were sacrificed and the accumulation and activation of CD8α^+^ DCs in the liver was determined (Figure 6A). We found that challenge with Inf. Spz promoted significantly higher accumulation of CD8α^+^ DCs population (*p* < 0.05) in the liver of primed mice compared to that of RAS challenge (Figure 6B).

Subsequently, the evaluation of activation status of CD8α^+^ DCs suggests that the pattern of difference between the two challenged groups with regard to the population having MHC-II^hi^ (Figure 6B) is very similar to the naive mice inoculated with Inf. Spz or RAS (Figure 5). However, % of MHC-II^hi^CD8α^+^ DCs expressing CD80 (1.54 ± 0.17%, Inf. Spz challenge vs. 0.21 ± 0.07%, RAS challenge, *p* < 0.05), CD86 (1.42 ± 0.19%, Inf. Spz challenge vs. 0.35 ± 0.04%, RAS challenge, *p* < 0.05), or CD40 (1.43 ± 0.42%, Inf. Spz challenge vs. 0.15 ± 0.03%, RAS challenge, *p* < 0.05) in the liver of Inf. Spz challenged mice were found much higher (Figure 6) compared to that of direct inoculation (Figure 5). Further, we found that Inf. Spz challenge induced enhanced expression of CD80 (MFI = 59,675 ± 1,524, Inf. Spz challenge vs. MFI = 46,113 ± 991, RAS challenge, *p* < 0.05), CD86 (MFI = 22,793 ± 651, Inf. Spz challenge vs. MFI = 16,406 ± 260, RAS challenge, *p* < 0.05) and CD40 (MFI = 20,380 ± 1,277, Inf. Spz challenge vs. MFI = 13,288 ± 112, RAS challenge, *p* < 0.05) (Figure 6C).

### Infectious Sporozoite Challenge Promotes CD4^+^ T Cell Activation and Augments RAS-Induced CD8^+^ T Cell Responses

**Figure 7:**
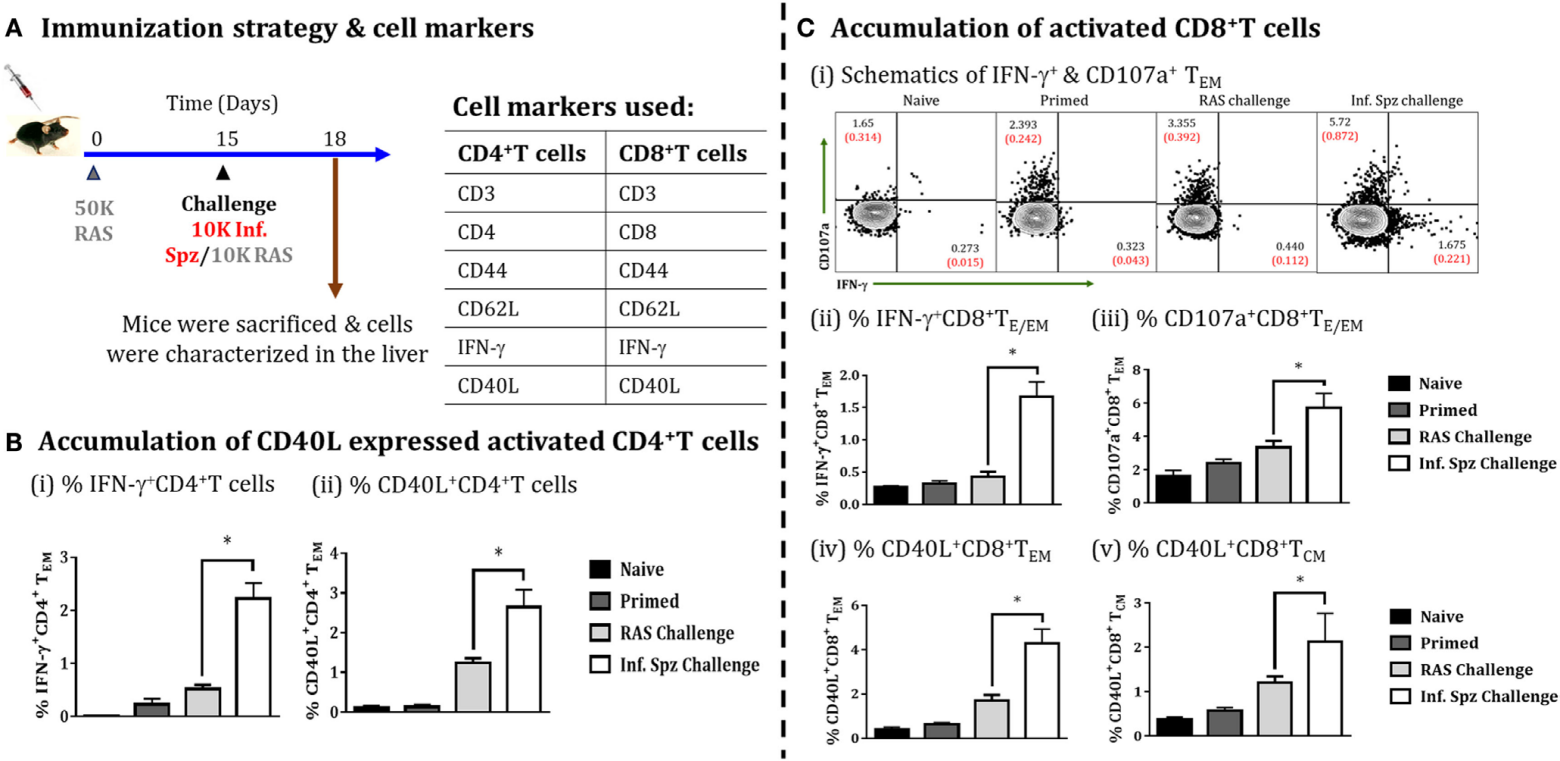
Accumulation of activated CD4^+^ T cells and CD8^+^ T cells in the liver on challenging the immunized mice, **(A)** immunization strategy and cell markers used to characterize CD4 and CD8 T cells. Mice were primed with 50K radiation-attenuated sporozoites (RAS), on day 15 one group of mice were challenged with 10K Inf. Spz and another group with 10K RAS. Mice were sacrificed 72 h post-challenge, **(B)** accumulation of activated CD4^+^ T cells in liver, (i) % IFN-γ-producing CD4^+^ T_E/EM_ cells, (ii) % CD40L expressed CD4^+^ T_E/EM_ cells. **(C)** Accumulation of activated CD8^+^ T cells, (i) schematics of IFN-γ^+^ and CD107a^+^ CD8^+^ T_E/EM_ cells, (ii) % IFN-γ-producing CD8^+^ T_E/EM_ cells, (iii) % CD107a expressed CD8^+^ T_E/EM_ cells, (iv) % CD40L expressed CD8^+^ T_E/EM_, (v) % CD40L expressed CD8^+^ T_CM_. Each bar shown in graph is mean of four individual mice per group. Percent cell shown is the percent out of total mononuclear cells or lymphocytes. *N* = 4 mice per group. Data are the mean ± SEM. The data were analyzed with non-parametric Mann–Whitney *U*-test. *p* < 0.05 is considered as a significant. Data shown represent one of two independent experiments.

The role of CD40 and CD40L (CD154) in APC licensing is well established (47). Moreover, CD4^+^ T cells *via* APC licensing is known to help CD8^+^ T cells *via* CD40 signaling and promotes CD8^+^ T cell activation and memory formation by providing the survival signal to T cells (48). We wanted to find out whether Inf. Spz challenge would induce the expression of CD40L in activated CD4^+^ T cells (Figure 7A). We looked for IFN-γ production and CD40L expression on the surface of activated CD4^+^ T cells in the liver in response to RAS or Inf. Spz challenge as part of the above experiment. We found significantly higher % of IFN-γ producing CD4^+^ T cells in the liver of Inf. Spz challenge mice to that of RAS challenge mice (2.23 ± 0.29% Inf. Spz challenge vs. 0.545 ± 0.075% RAS challenge, *p* < 0.05) (Figure 7Bi); also the % of CD40L expressed cells was significantly higher with at least two folds higher expression on the surface of CD4^+^ T cells of Inf. Spz challenge group mice (2.65 ± 0.43%, Inf. Spz challenge vs. 1.24 ± 0.12%, RAS challenge, *p* < 0.05) (Figure 7Bii). We also found that the effector functions of CD8^+^ T cells in response to infectious sporozoite challenge were significantly improved compared to RAS challenge (Figure 7C). It is worth noting that both type of challenge induced cytotoxic activity (CD107a) (5.72 ± 0.88%, Inf. Spz challenge vs. 3.36 ± 0.39%, RAS challenge, *p* < 0.05), while infectious sporozoite challenge specifically promoted IFN-γ response among CD8^+^ T cells (1.67 ± 0.22%, Inf. Spz challenge vs. 0.43 ± 0.08%, RAS challenge, *p* < 0.05) (Figure 7Cii,iii). Further, we have analyzed the CD40L expression on the surface of CD8^+^ T cells (T_E/EM_ and T_CM_) in response to Inf. Spz challenge in liver. While 4.3 ± 0.62% CD8^+^ T_E/EM_ cells in the liver of RAS primed mice expressed CD40L upon Inf. Spz challenge, the same was found to be 1.36 ± 0.39% following RAS challenge. The expression of CD40L on CD8^+^ T_CM_ population in the livers of mice followed the similar trend (*p* < 0.05) (Figure 7Civ,v).

## Discussion

Dendritic cells play a central role in the initiation of adaptive immune responses by efficient presentation of antigens to T cells. The presence of numerous surface and IC receptors capable of sensing microbial components and inflammation prepares the DCs for their function. The ability of DCs to regulate adaptive immunity primarily depends on the state of maturation. The microenvironment created at the site of infection largely influences the maturation of DCs following antigen uptake. The process of activation and maturation of DCs is reported to promote higher expression of MHC and costimulatory molecules on their surface (49, 50). The infectious status of pathogens is known to induce activation and maturation of DCs leading to the efficient antigen presentation and T cell priming. In WSV model, DCs are shown to be responsible for the generation of LS-specific CD8^+^ T cells that confer the protective immunity (25–27). While DCs pulsed with RAS have been shown to generate antigen-specific CD8^+^ T cell, the adoptive transfer of DCs loaded with CSP antigen leading to generation of antigen-specific CD8^+^ T cells and conferring protection against sporozoite challenge provides the clues that DCs are responsible APCs for inducing sterile immunity (51). It was further supported from the depletion studies in which mice fail to activate LS-specific CTLs in response to RAS immunization (24). Here, we focused on the specific subset of DCs, i.e., CD8α^+^ DCs because of its critical role in inducing LS-specific CD8^+^ T cells required for sterile protection (25–27).

The RAS vaccination is very promising in inducing sterile protection, but relatively short lived. A recent study suggests that repeated immunizations with late arresting GAP provide sterile protection that extends beyond one year after the last immunization (10). However, the level of protection is partial (20–40%). In our study, we demonstrated the complete sterile protection up to 18 months in RAS immunized mice challenged with Inf. Spz (11). In the present study, we have shown that challenging the immune host with Inf. Spz brings the qualitative changes in RAS-induced LS-specific CD8^+^ T cells associated with longer-lived protection (Figure 1). We envision that the infectious status of sporozoites play a critical role in bringing qualitative changes in LS-specific CD8^+^ T cells possibly by modulating the innate immune responses. The innate immune response decides the fate of adaptive immunity and the nature of innate immune response is determined by the kind of danger signals host perceives during the onset of infection or vaccination. RAS is known to be arrested at early stage of its developmental cycle, while Inf. Spz develops until the late stage causing extensive damage in the liver. It is possible that the profiles of pathogen-associated molecular patterns and or danger-associated molecular patterns engaged by the host in case of Inf. Spz challenge would be different, and the nature of innate immune response generated would be different from that of RAS. Here, we determined the activation status of CD8α^+^ DCs to understand the effect of differential host response/innate immune response induced by the infectious status of the sporozoites (39).

This has been demonstrated that CD8α^+^ DCs are responsible for the activation and induction of malaria LS-specific CD8^+^ T cells in response to RAS vaccination and confers the protective immunity in the liver (25, 26). In the present study, we found that this specialized population of DCs, known for antigen cross-presentation, was accumulated at least three times higher in the liver of mice that received Inf. Spz. Our results are consistent with the others as CD8α^+^ DCs increased in number in the livers of mice immunized with RAS or GAP (25, 26). As the accumulation of CD8α^+^ DCs in the liver was thought to be the result of migration of the same from circulation, the expression of chemokines (CCL-20 and CCL-21) in the liver of mice was measured. The higher expression of the chemokines in mice inoculated with Inf. Spz. suggestive of the fact that greater accumulation of CD8α^+^ DCs at the site of infection is because of the infectious status of the sporozoite (Figure 3D).

However, higher accumulation may not be always correlated with qualitatively better T cell response. The ability of DCs to modulate T cells is primarily dependent on their activation status, which involves the upregulation of MHC molecules and increased expression of costimulatory molecules (CD80, CD86, and CD40) on the surface of DCs (52, 53). Hence, we examined the role of infectious status of sporozoites in modulating CD8α^+^ DCs. Our results, in fact, suggest that expression levels of MHC and costimulatory molecules induced by Inf. Spz meet the requirements for better T cell activation and memory formation. It is known that signal-2, i.e., CD80 or CD86 besides MHC/peptide complex (signal-1) is essential for the activation of T cells (50, 52, 53). Similarly, CD40-CD40L signaling is known to be involved in cross-presentation and APC licensing (54, 55) to promote survival of antigen-specific CD8^+^ T cells (26, 56, 57). Therefore, Inf. Spz inoculation/challenge that enhances not only the accumulation of CD8α^+^ DCs in liver, but also favored generation of higher frequencies of activated CD4^+^ T cells with greater expression of CD40L. This in turn might promote APC licensing of CD8α^+^ DCs and provide help to CD8^+^ T cells. The expression of CD40L on the surface of varieties of cells including B cells, NK cells, basophils, mast cells, and antigen-specific CD4^+^ T cells. These cells are predominantly responsible for the activation of CD40 expressing cells, particularly APCs that is essential for cross-priming of functional CD8^+^ T cells (47).

It is known that microenvironment at the site of infection plays a critical role in determining the state of APCs (36, 38, 58). Therefore, we made attempt to understand whether microenvironment in liver created following sporozoite inoculation or challenge could be influenced by the infectious status of sporozoite. While the high levels of chemokine expression were found to be regulated by the infectious status of sporozoite, TLR signaling promoting IRF-7 and type I interferons, particularly IFN-α expression was favored by the Inf. Spz (Figures 2B and 4D). The type I interferons, major inflammatory cytokines during initiation of immune response are critical for bridging innate and adaptive immune response (59). The key functions of type I IFN response involve activation and differentiation of DCs by promoting the expression of MHC molecules and various costimulatory molecules for the priming of T cells (34, 60). Furthermore, type I interferons promote the cross-presentation of antigen to the CD8^+^ T cells (47, 61) and are known to favor formation of memory CD8^+^ T cells (62). As CD8α^+^ DCs are preferred APCs for cross-presentation, it is possible that the Inf. Spz through the TLR signaling and interferons better prepares them to differentiate the LS-specific CD8^+^ T cells to become memory.

Clearly, these results demonstrate a role of Inf. Spz challenge in accumulation and activation of CD8α^+^ DCs. The CD8α^+^ DCs accumulated and activated at the site of infection, i.e., the liver in response to sporozoites and may take up LS antigen from the apoptotic hepatocytes. In our results, higher expression of various immune mediators in case of Inf. Spz inoculation reflects the key role of innate immune response in shaping up the CD8^+^ T cell immunity.

The expression of CD40 or CD40L on CD8^+^ T cells promoted by Inf. Spz challenge provides us a plausible explanation that CD8^+^ T cells either receive signals from the licensed CD8α^+^DCs through CD40-CD40L or from CD4^+^ T cells through CD40L-CD40 or from both to become qualitatively better memory T cells. Our data generated from whole transcriptome analysis supports the idea that infectious status of sporozoite promotes MHC-II mediated response (Figure 7B), a critical requirement for APCs to be licensed (48). Our data, for the first time, formally show the role of infectious status of sporozoites in orchestrating qualitative changes among the RAS-induced CD8^+^ T cell responses, critical for protracted protection against *Plasmodium* infection. This study provides clues for the nature of innate immune responses that a protective malaria vaccine would require. The most important parameters include accumulation and activation of CD8α^+^ DCs at site of infection which is crucial for long-lasting immune responses against LS *Plasmodia* infection. Further, the production of type I interferon and induced signaling appear to shape protective CD8^+^ T cell response. In conclusion, our study might help to find out the immunological determinants for the generation of long-lived protective immunity.

## Ethics Statement

All animal experiments were reviewed and approved by the Institutional Animal Ethical Committee (IAEC) of Nirma University (CPCSEA Reg. No: 883/PO/ReBi/S/05/CPCSEA).

## Author Contributions

SD, Rajesh P, HP, and NY: designed the work or the acquisition, analysis, or interpretation of data for the work, and manuscript writing. Ritika P, KP, AM, VB, and UJ: assisted in experimental work.

## Conflict of Interest Statement

The authors declare that the research was conducted in the absence of any commercial or financial relationships that could be construed as a potential conflict of interest.

## Footnotes

^1^http://www.ncbi.nlm.nih.gov/sra/SRP129582.

^2^http://www.uniprot.org/.

^3^http://pantherdb.org/.

^4^http://www.kegg.jp/.

^5^http://www.kegg.jp/kegg/tool/map_pathway1.html.

## Supplementary Materials

Figure S1 Schematics for gating strategy used for flow data analysis to characterize CD8^+^ T cells by using CD3 and CD8 cell surface marker on gated lymphocytes population and further characterized the CD8^+^ T cells into T central memory (T_CM_; CD44^+^CD62L^+^) and T effector memory (T_EM_; CD44^+^CD62L^−^) with IFN-γ and CD107a expression.

Figure S2 **(A)** Heat map showing the expression changes between Sham, radiation-attenuated sporozoites (RAS), and infectious sporozoites (Inf. Spz) inoculated mice livers, expressed as the log2 of normalized. It shows the significantly expressed genes (<1-fold change) between the Sham and Inf. Spz group and their gene expression scales for lighter to darker. **(B)** Validation of expression of the genes related to toll-like receptor (TLR) and interferon signaling pathway in mouse liver following RAS and Inf. Spz Inoculation through qPCR analysis.

Figure S3 Schematics for gating strategy used for flow data analysis to characterize different population of CD8α^+^ dendritic cells (DCs) one the basis of MHCII expression and further with the expression of CD80, CD86, and CD40. The gating strategy of DCs analysis as per gating described in mononuclear cells (MNCs) gate defined by FSC-A/SSC-A plot taken from singlet population (singlets were described from FSC-A/FSC-H plot). Briefly, lymphocytes (NK cells and B cells) and debris were excluded by a SSC/FSC plot by means of a MNCs gate including DCs.

## Abbreviations

RAS, radiation-attenuated sporozoites; Inf. Spz, infectious sporozoites; LS, liver-stage; DCs, dendritic cells; APC, antigen-presenting cells.
