# Supplementary figures and images for: Infectious Sporozoites of Plasmodium berghei Effectively Activate Liver CD8α+ Dendritic Cells
Source: Front Immunol. 2018 Feb 8;9:192. doi: 10.3389/fimmu.2018.00192 (PMC5809440; doi:10.3389/fimmu.2018.00192)

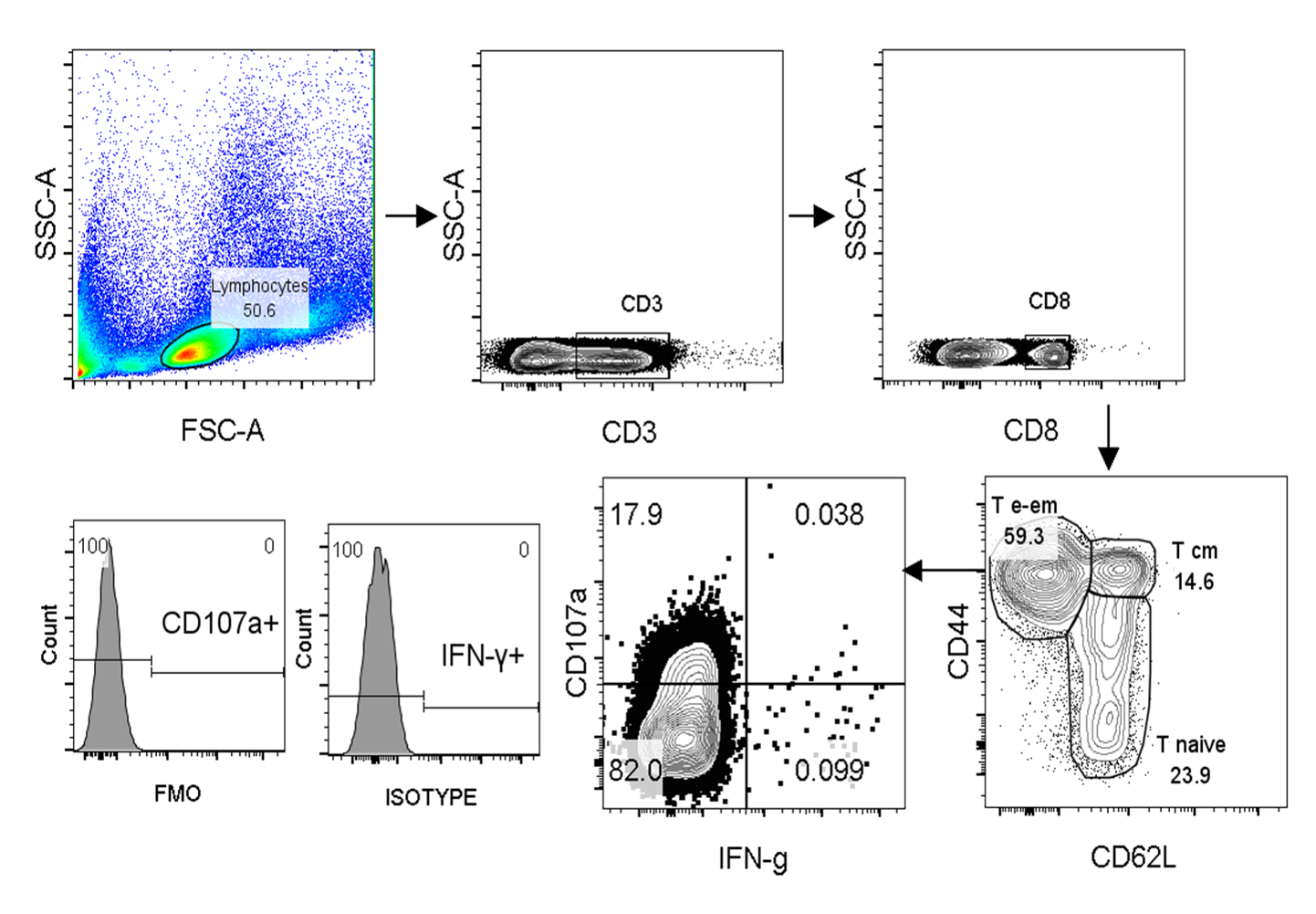

Supplement: Figure S1 — Schematics for gating strategy used for flow data analysis to characterize CD8+ T cells by using CD3 and CD8 cell surface marker on gated lymphocytes population and further characterized the CD8+ T cells into T central memory (TCM; CD44+CD62L+) and T effector memory (TEM; CD44+CD62L−) with IFN-γ and CD107a expression. [file Image_1.tif]

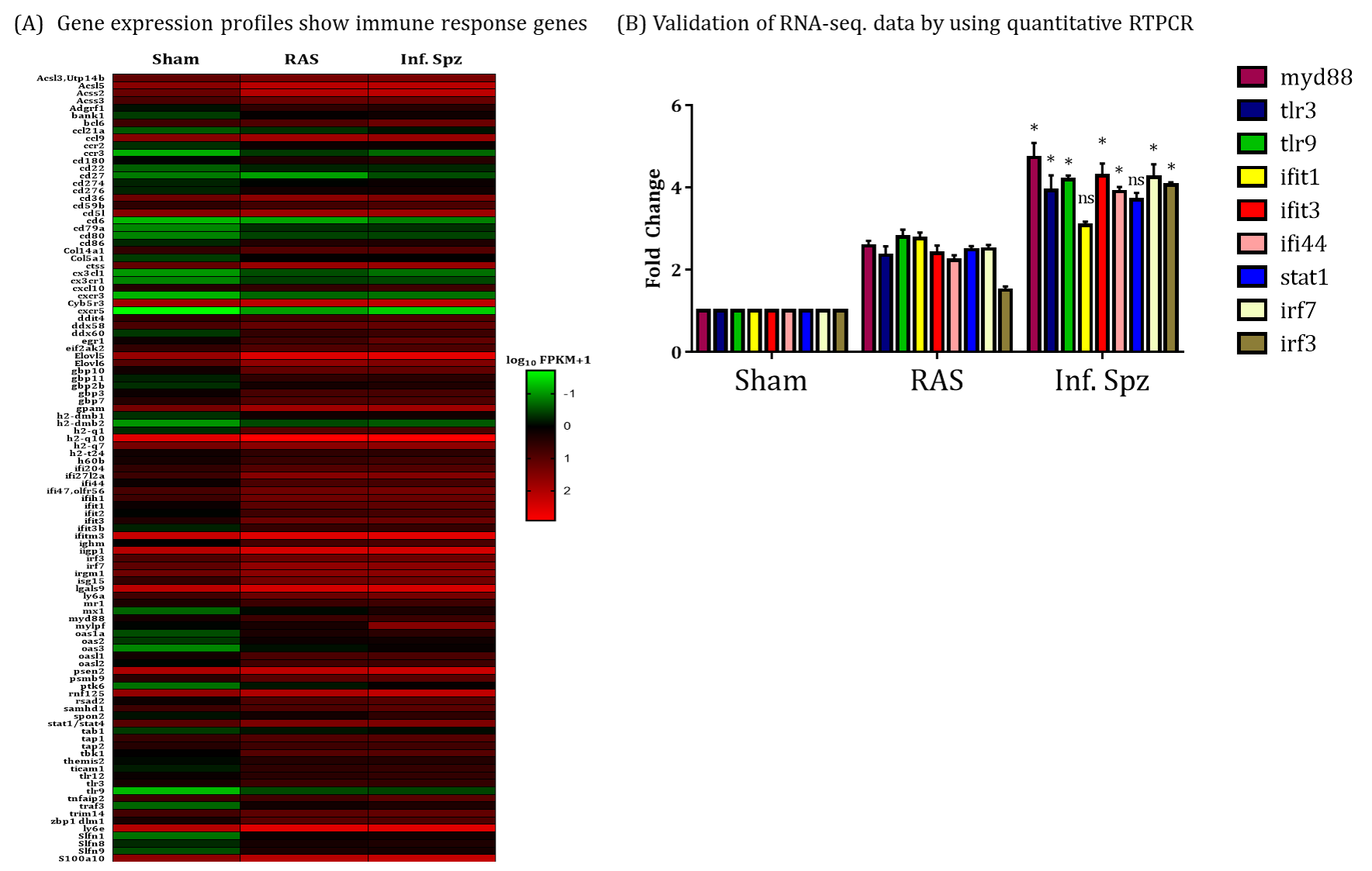

Supplement: Figure S2 — (A) Heat map showing the expression changes between Sham, radiation-attenuated sporozoites (RAS), and infectious sporozoites (Inf. Spz) inoculated mice livers, expressed as the log2 of normalized. It shows the significantly expressed genes (<1-fold change) between the Sham and Inf. Spz group and their gene expression scales for lighter to darker. (B) Validation of expression of the genes related to toll-like receptor (TLR) and interferon signaling pathway in mouse liver following RAS and Inf. Spz Inoculation through qPCR analysis. [file Image_2.tif]

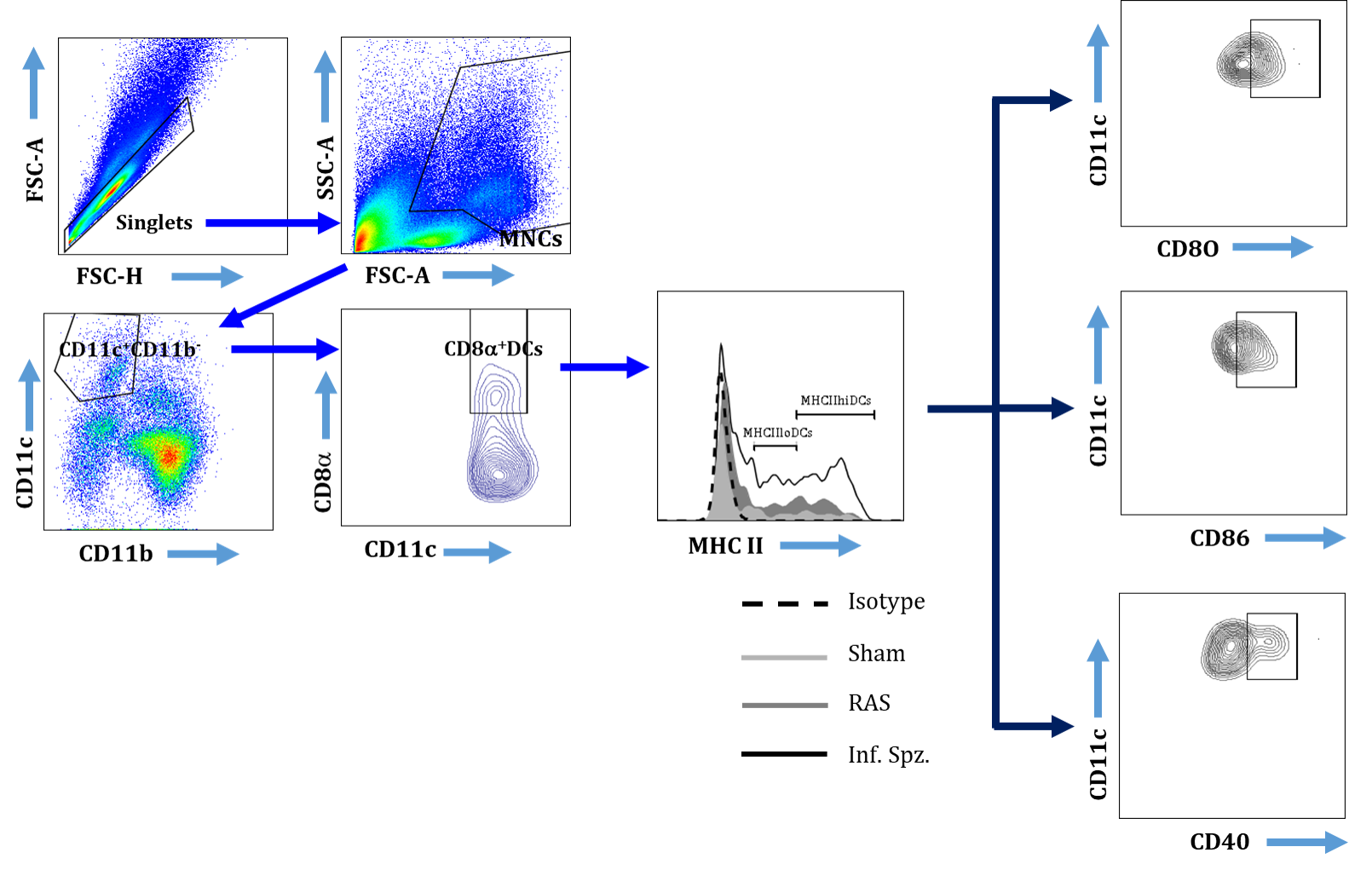

Supplement: Figure S3 — Schematics for gating strategy used for flow data analysis to characterize different population of CD8α+ dendritic cells (DCs) one the basis of MHCII expression and further with the expression of CD80, CD86, and CD40. The gating strategy of DCs analysis as per gating described in mononuclear cells (MNCs) gate defined by FSC-A/SSC-A plot taken from singlet population (singlets were described from FSC-A/FSC-H plot). Briefly, lymphocytes (NK cells and B cells) and debris were excluded by a SSC/FSC plot by means of a MNCs gate including DCs. [file Image_3.tif]
